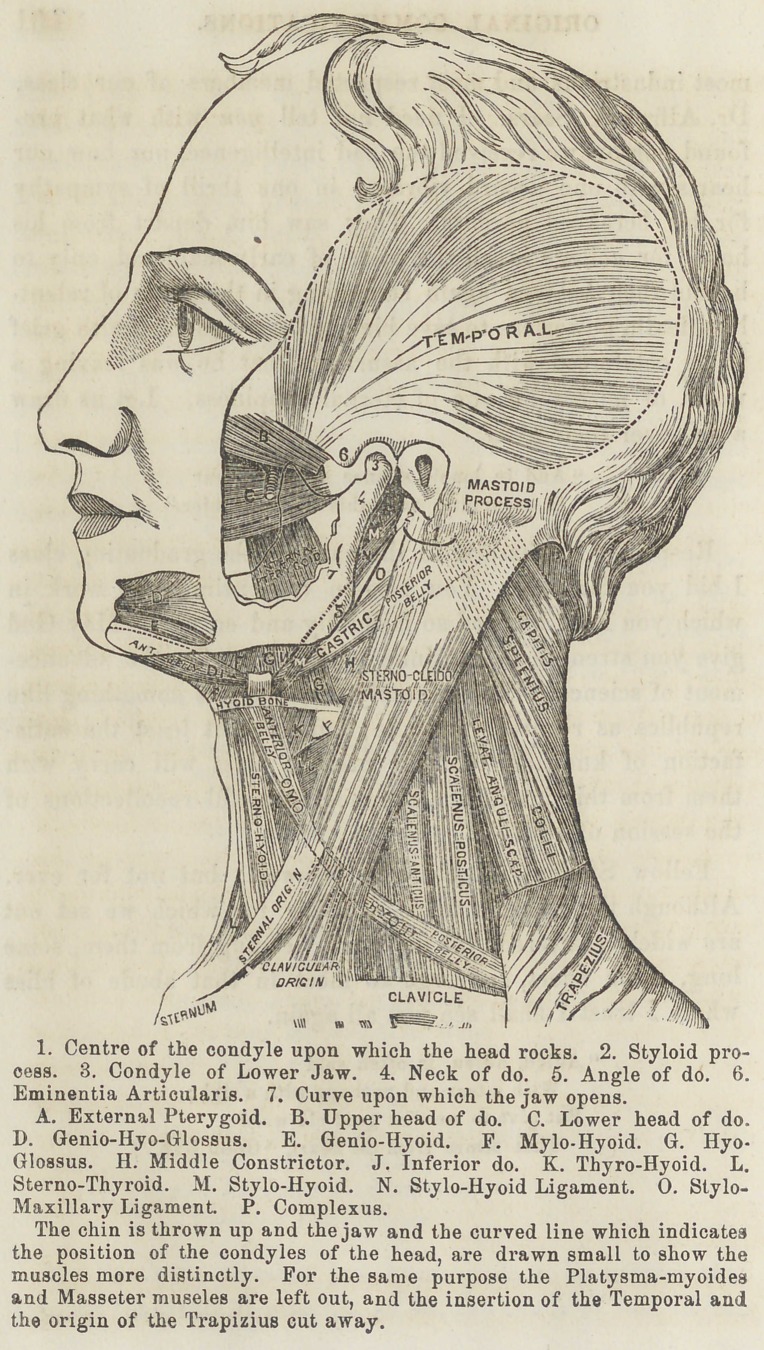# On the Physiological Action of the Muscles Concerned in the Movements of the Lower Jaw

**Published:** 1868-03

**Authors:** 


### ON THE PHYSIOLOGICAL ACTION OF THE MUSCLES CONCERNED IN THE MOVEMENTS OF THE LOWER JAW.

[We give place to the following from an essay by Dr. T. B. Gunning, on the Muscles of the Head, Neck, Jaw, and Palate. Regarding it as one of the best papers upon the points embraced, that has ever been written, we bespeak for it a very thorough and critical reading. The remaining part of the essay upon the subject here indicated will appear in our next number.]—Ed.

The necessity for muscles of great power, and acting upon long levers, to turn the head quickly, is demonstrated by the action of the sterno-cleido-mastoid. In very quick turning of the head the muscle acts instantaneously; this, however, is but seldom. In the ordinary rotation of the head, it takes no part whatever, unless the head is obstructed as when lying down on the side. But if the head is turned far around, the muscle always acts firmly in the latter part of the movement. This can be verified if the body is held upright and the forefinger placed in the interclavicular notch, with the thumb and second finger resting on the tendons of the muscles, and notice taken of the tightening and relaxation of the tendons. The comparative indifference of this muscle to the head's rotation can be more easily demonstrated in the evening, or when fatigued. From this it appears that the action of the *sterno-mastoid* in turning the head is of a very secondary character. It acts only when the rotators which pass from the axis to the atlas and occipital bone, and the *splenius capitis* and *colli* of the opposite side, are already in action, and even then only to assist in turning the head quickly, to carry it further round, or to overcome obstruction. When it assists in turning the head it draws one mastoid process forward, while the splenius pulls the other mastoid process backward.

The sterno-cleido-mastoid muscle is said to be a rotator, a flexor, and an extensor of the head. What this flexing of the head means, in addition to lateral movement, may be learned by the following quotation: "The sterno-mastoid muscles, when both are brought into action, serve to depress the head upon the neck, and the neck upon the chest."* These views are also maintained by J. Cruveilhier† and may be accepted as those not only of the French anatomists and physiologists generally, but also of the German and English with few exceptions. Professor Henle, however, says positively that these muscles do not flex the head down in front, and that they lift the head and bend the neck when the body is brought up in rising from the back.‡ This is a great advance upon what is said by others, but beyond this he gives no intimation of understanding their peculiar and most important function.

The insertion of the sterno-cleido-mastoid muscle is around the front of the mastoid process, and back along the superior curved line, about half the distance between the mastoid process and the centre of the occipital protuberance, while the front of the mastoid process is nearly always on a line with the centre of the condyles of the occipital bone (in rare instances, however, it is nearer the front of the condyle). The sterno-cleido-mastoid muscle is consequently inserted back of the centre upon which the head rocks (except in rare cases when a small portion of the muscle is a little forward of it). Notwithstanding this, it is set down as rocking the head forward, and the action of the muscle in rising is brought forward to prove it. This experiment, however, if properly conducted and explained, will prove the contrary. If the experimenter, while lying flat on his back, with the forefinger resting in the interclavicular notch, and the thumb and second finger on the tendons, will raise his head and shoulders a little, he will

---

*Gray's Anatomy. 2d Amer. edit. p. 256. Phil. 1865.

†Traité d'Anatomie Descriptive. Troisieme edit., tome deuxieme, p. 173, Paris. 1851.

‡Handbuch der Muskellehre des Menschen von Dr. T. Henle, Professor der Anatomie in Goettingen. (Page 110).

find that the muscles are acting strongly; then by staying in that position and rocking the head backward and forward, it will be felt that the muscles are unaffected in any part of their fibres, and that they pay no attention to the movement of the head, neither the tendons on the sternal portions nor those on the clavicular being relaxed for a moment. Then sit up, throw the head forward sufficiently to relax the tendons, and rock the head as before; it will now be found that the tendons remain relaxed, showing that tightness of the tendons did not conceal action of the muscles in the first experiment, and demonstrating that the *sterno-cleido-mastoid* muscles do *not* "serve to depress the head upon the neck." In bringing the head forward these muscles act only until the head comes to its centre of balance, when the tendons relax and remain so, even when the chin touches the breast. But if the head is obstructed in this downward movement, these muscles will then assist to bring it down in front and to hold it there. The sterno-cleido-mastoid muscles do not, however, in this rock the head upon the atlas, but bring and keep the atlas forward. Neither are they "extensors of the head" in the sense indicated by the books, which seems at first sight to accord more with their insertion back of the centre of the condyles. But the insertion is so peculiar that it requires consideration to determine how the muscles affect the head. The mastoid process is always below the superior curved line upon which the back part of the muscle is inserted. When the process is large it may be more than an inch below it, although much less when the process is small, as in childhood before the cells are developed. Moreover, the uniformity of position between the mastoid processes and the condyles horizontally is not met with in their vertical relation, the condyles being on some skulls more than half an inch lower than the mastoid processes, while on others the processes are as much below the condyles, the large proportion being between these extremes. These variations go far to show that the sterno-mastoid muscles are not intended to rock the head backward, for when the mastoid process is much lower than the condyles, and especially when it is large and projects forward somewhat, to correspond to the direction of the muscle it follows that as the head is pulled downward (by the *trapezii*, &c.,) the mastoid processes go upward and forward; consequently if the *sterno-cleido-mastoid* were to act to bring the head down behind, the portion on the mastoid process — the strongest part of the muscle — would hold the head down in front, probably as much as that on the occipital bone would pull it down behind. But their action can be tested by lying down so as to remove the necessity for action of the muscles to hold the atlas. In this position (care being taken not to lift the atlas, or neck) the *sternal* portion of the muscles will not act in concert with the other muscles to rock the head back, even if the whole weight of the body is thrown upon the back of the head, and I have been unable to find any action in the clavicular portion, although the action of this part of the muscle is so delicate and prompt that it can be distinctly felt when the foot is raised in walking, the head and body being then thrown over the other side to restore the balance. Further, when the *sterno-cleido-mastoid* and the *splenius* of the same side are acting in concert to pull the head down to the shoulder, no backward movement of the head is discoverable. *This* is conclusive, for both these muscles having similar insertions, if one rocks the head back the other must, and their combined action would be manifest if they exerted it.

It has been previously shown that this muscle acts as a *rotator* only by sometimes assisting the *splenius*, &c., of the opposite side, and as a lateral *flexor*, in connection with the *splenius* of the same side, but only when the head is obstructed, and then generally by its clavicular portion, the sternal acting only in extreme necessity. It is now seen that it does not flex the head down in *front*, that is upon the atlas, and that its action as an *extensor* of the head can not be demonstrated. The proper function of the *sterno-cleido-*

*mastoids* when acting in concert, is to give anterior support to the top of the spine, the *splenii* muscles giving posterior support. This may be easily proved by sitting down and watching the tendons. When the head is back of its centre of support both the sternal and clavicular tendons are tightened, when rising they become tenser until the head is started, as it comes into balance they relax. On sitting down, the tendons tighten to check the head as it goes back out of balance. Sudden forward movements tighten them until the head is in motion, they then slacken as the head is forward of the centre and the atlas supported by the *splenii* muscles. If the head is in balance, any pressure upon the forehead acts with increased force upon the atlas and brings the muscles into action to keep it upright. The action of the sterno-cleido-mastoid muscles in these movements is but a modification of the service rendered by them in raising the head from the horizontal position, in doing which the muscles at first support more than the weight of the head, for in supporting the mastoid processes they support the atlas, and make it a fulcrum between the bulk of the head and the counter-balance at the other end of the lever, but as the body comes upright and the head into balance, the strain upon the sterno-mastoid muscles gradually diminishes, until the head is held by the posterior muscles, when the atlas bears all the weight *vertically*.

[A reference to the figure will render this explanation more apparent. The same figure also illustrates the action of the muscles of the lower jaw, and confirms the opinions expressed in the subsequent portions of this paper.]

The hyoid bone, in addition to the muscles which pass to it from parts above the lower border of the jaw, gives attachment to others, which pass up the front of the neck below the jaw. Of these the *sterno-thyroid* arises close to the centre of the posterior surface of the upper bone of the sternum, and falling back somewhat as it passes up, is inserted into the side of the thyroid cartilage, from whence the *thyro-hyoid* (appearing like a continuation of the preceding) goes up and is inserted into the body and greater cornu of the hyoid bone. The *sterno-hyoid* arises from the sternum and end of the clavicle and is inserted into the lower border of the body of the hyoid bone. It is separated considerably from its fellow at its origin, but crosses the sterno-thyroid and approaches it in the middle of its course; it leaves the front of the thyroid cartilage uncovered.

The *omo-hyoid* arises from the upper border of the scapula, and occasionally from the transverse ligament which crosses the supra-scapular notch. It passes across and up the side of the neck to be inserted into the body of the hyoid bone. It crosses under the *trapezius* and *sterno-cleido-mastoid* muscles but over the *scaleni* and *thyro-hyoid.* It is a double-bellied muscle united by a tendon which is held down by a process of the deep cervical fascia. The first portion is nearly horizontal in its course, but underneath the sterno-mastoid muscle, where the cervical fascia passes around the tendon, it turns up so that the second portion is nearly vertical in its course to the hyoid bone. These are the directions of the muscle when at rest, but when active it approaches the line of its attachments and the cervical fascia is drawn upward and backward.

The *digastric*, another double-bellied muscle, has peculiar relation with the preceding. It arises from the digastric notch, on the inner side of the mastoid process of the temporal bone, and passes downward, forward, and inward, to the side of the hyoid bone, where its rounded tendon (after passing through the stylo-hyoid muscle) is held by an aponeurotic loop in connection with the side of the body of the hyoid bone above the insertion of the omo-hyoid. The muscle then passes forward and is inserted into a large depression on the inner side of the lower border of the jaw close to the symphysis. The tendon which divides the posterior and longer belly from the anterior, gives off a large aponeurotic layer, which is attached to the body and great cornu of the hyoid bone; and with the portion on the opposite side is termed the *supra-hyoid-aponeurosis*, which forms a strong layer of fascia between the anterior portions of the two muscles, and a firm investment for the other muscles of this region. The *digastric* muscle is peculiar in not being inserted into the hyoid bone, but attached to it by a loop; this allows the muscle to act without interfering too much with the hyoid bone. The muscle has not, however, that freedom which is attributed to it as a reflected cord, for its aponeurotic connection with the hyoid bone and adjoining muscles prevents it from sliding through the loop which attaches it to the hyoid bone, except to a very limited extent. This powerful muscle exerts great influence from the various and important movements in which it takes part.

The last muscle to be described in this connection, the *platisma myoides*, is very distinctly separated from all the others. It is a broad thin plane of muscular fibres, immediately beneath the skin, on the side of the neck. It arises from the clavicle and acromium, and from the fascia covering the upper part of the pectoral deltoid and trapezius muscles, and going upward and forward, it covers in the angle and the border of the jaw to the symphysis. It is inserted into the lower border of the jaw, in front, but back of the commissure of the lips it is found interlaced with the muscles above. It affords muscular support to the integument, and a cover to the muscles beneath, but leaves the thyroid cartilage and the front of the trachea free.

[TO BE CONTINUED.]

## Figures and Tables

**Figure f1:**